# Short‑term outcomes of minimally invasive endoscopic onlay repair for diastasis recti and ventral hernia repair: a systematic review and meta‑analysis

**DOI:** 10.1007/s00464-025-11555-1

**Published:** 2025-02-07

**Authors:** Francesco Brucchi, Luigi Boni, Elisa Cassinotti, Ludovica Baldari

**Affiliations:** 1 University of Milan, 20122 Milan, Italy; 2 Department of General and Minimally-Invasive Surgery, Fondazione IRCCS Ca’ Granda Ospedale Maggiore Policlinico, Via Francesco Sforza 35, 20122 Milan, Italy; 3 Department of Pathophysiology and Transplantation, University of Milan, Via Francesco Sforza 35, 20122 Milan, Italy; 4 Department of Clinical Sciences and Community Health, University of Milan, Via della Commenda 19, 20122 Milan, Italy

**Keywords:** Laparoscopic/endoscopic, Onlay, ENDOR, Diastasis recti, Ventral hernia, Umbilical hernia

## Abstract

**Background:**

Endoscopic onlay repair (ENDOR) approach is gaining traction as a promising technique for the treatment of diastasis recti and associated ventral hernia. However, comprehensive evidence regarding its perioperative and short-term outcomes remains scarce. The objective of this meta-analysis is to provide a comprehensive summary of the existing evidence concerning perioperative and short-term postoperative outcomes.

**Methods:**

A systematic literature review was reported according to the Preferred Reporting Items for Systematic Reviews and Meta-Analyses (PRISMA). A comprehensive search was conducted in MEDLINE, Embase, and CENTRAL until August 1st, 2024. Articles reporting outcomes of ENDOR in adult population diagnosed with diastasis recti associated or not with primary or incisional ventral hernia were included. Primary outcomes were evaluated based on safety and short-term measures, including intraoperative and short-term postoperative characteristics. A fixed effects model was used for meta-analysis. The methodological quality of the studies was assessed using the Methodological Index for Non-randomized Studies (MINORS) criteria.

**Results:**

A total of 12 studies (480 patients) were identified. Minimally invasive ENDOR resulted in an intraoperative complications and conversion rate of 1.0% [95% CI 0.0–2.0%]. The rate of seroma was 25% [95% CI 12.0–39.0%], the one of surgical site infection was 1% [95% CI 0.0–2.0%] and the rate of hematoma was 2% [95% CI 1.0–3.0%]. After a median follow-up of 16 months (1.8–39), the rate of recurrence was 2% [95% CI 0.0–3.0%].

**Conclusions:**

The minimally invasive ENDOR approach stands out as a safe and effective method for diastasis recti and associated ventral hernia repair in selected patients, exhibiting low rates of intraoperative complications and yielding favorable outcomes.

Prospero registry

Registration number: CRD42024573235

**Supplementary Information:**

The online version contains supplementary material available at 10.1007/s00464-025-11555-1.

Diastasis recti (DR) is a condition characterized by an increase of the normal distance between the rectus abdominis muscles, usually exceeding 2.2 cm. Patients presenting with DR typically fall into one of following profiles: middle-aged and elderly obese men or short and thin women after full-term pregnancies. DR often coexists with primary abdominal wall defects, such as umbilical hernias, epigastric hernias, or both, or with incisional hernias [1–3]. Primary defects and incisional hernias occurring alongside diastasis recti pose a complex challenge in the field of abdominal wall reconstruction [4, 5]. Traditional open sublay repair of midline ventral hernias facilitates convenient anterior diastasis recti plication, but entails cosmetically unattractive large incisions. Laparoscopic intraperitoneal mesh (IPOM) repair offers a minimally invasive alternative, but it requires an ergonomically unfavorable posterior approach for DR plication, that additionally results in the development of an unsightly fold of midline soft tissue [6].

Emerging minimally invasive surgical (MIS) techniques, including laparoscopic and robotic approaches, have been identified as promising alternatives for addressing diastasis recti, whether occurring independently or in conjunction with anterior abdominal wall defects. The retro-muscular placement of mesh and minimal fixation requirements in sublay repairs, such as the enhanced-view totally extraperitoneal approach (eTEP), Robotic Transabdominal Retromuscular Umbilical Prosthetic Hernia Repair (TARUP), and Mini- or Less-open Sublay Operation (MILOS) techniques, present notable advantages. However, the complexity associated with unfamiliar anatomical landmarks for general surgeons, coupled with the challenges of suturing the superior abdominal wall, represents a significant limitation of these procedures [7–9]. Nevertheless, intraperitoneal techniques like IPOM plus offer greater ergonomics, but they are associated with the risks of accessing the peritoneal cavity, such as injury of hollow viscera, and placing intraperitoneal prosthesis [10].

Over the past decade, a new surgical technique for DR and ventral hernia repair has been described in literature, with few variations and different definitions according to the authors: “pre-aponeurotic endoscopic repair” (REPA) [11]; “endoscopic-assisted linea alba reconstruction” (ELAR) [12]; “minimal invasive linea alba reconstruction” (MILAR) [13]; “subcutaneous videosurgery for abdominal wall defects” (SVAWD) [14]; “subcutaneous onlay endoscopic approach” (SCOLA) [15]. All these techniques pivot on anterior plication of the diastasis associated with prefascial mesh placement. Delving into the technique, the preaponeurotic space is dissected from the subcutaneous adipose tissue, from the lower abdomen to the costal margin, then the linea alba is plicated to repair the DR and the ventral hernia, in the end a mesh is placed on the preaponeurotic space [3, 15–17]. Furthermore, the exposure resulting from the extensive dissection enables additional wall defects’ diagnosis, allowing simultaneous repairs [17]. However, it is crucial to ensure that lateral dissection does not extend beyond 3 cm from the edge of the rectus abdominis to minimize the risk of skin ischemia and postoperative seroma formation [17]. In order to avoid suture tension and decrease postoperative pain, external oblique muscle release, either unilateral or bilateral, can be associated to the procedure.

Initial literature findings show favorable outcomes concerning recurrence rates, postoperative complications and patient satisfaction [15, 17]. However, robust evidence supporting this technique is still lacking. For ease of reference, the name of the technique will be standardized as endoscopic onlay repair (ENDOR) throughout the article, as proposed by Malcher et al. in 2021 [18]. The aim of this study is to perform a systematic review of the literature with meta-analysis to comprehensively summarize and evaluate the safety and short-term outcomes of the ENDOR procedure for diastasis recti and ventral hernia repair.

## Material and methods

### Data sources and searches

**Fig. 1:**
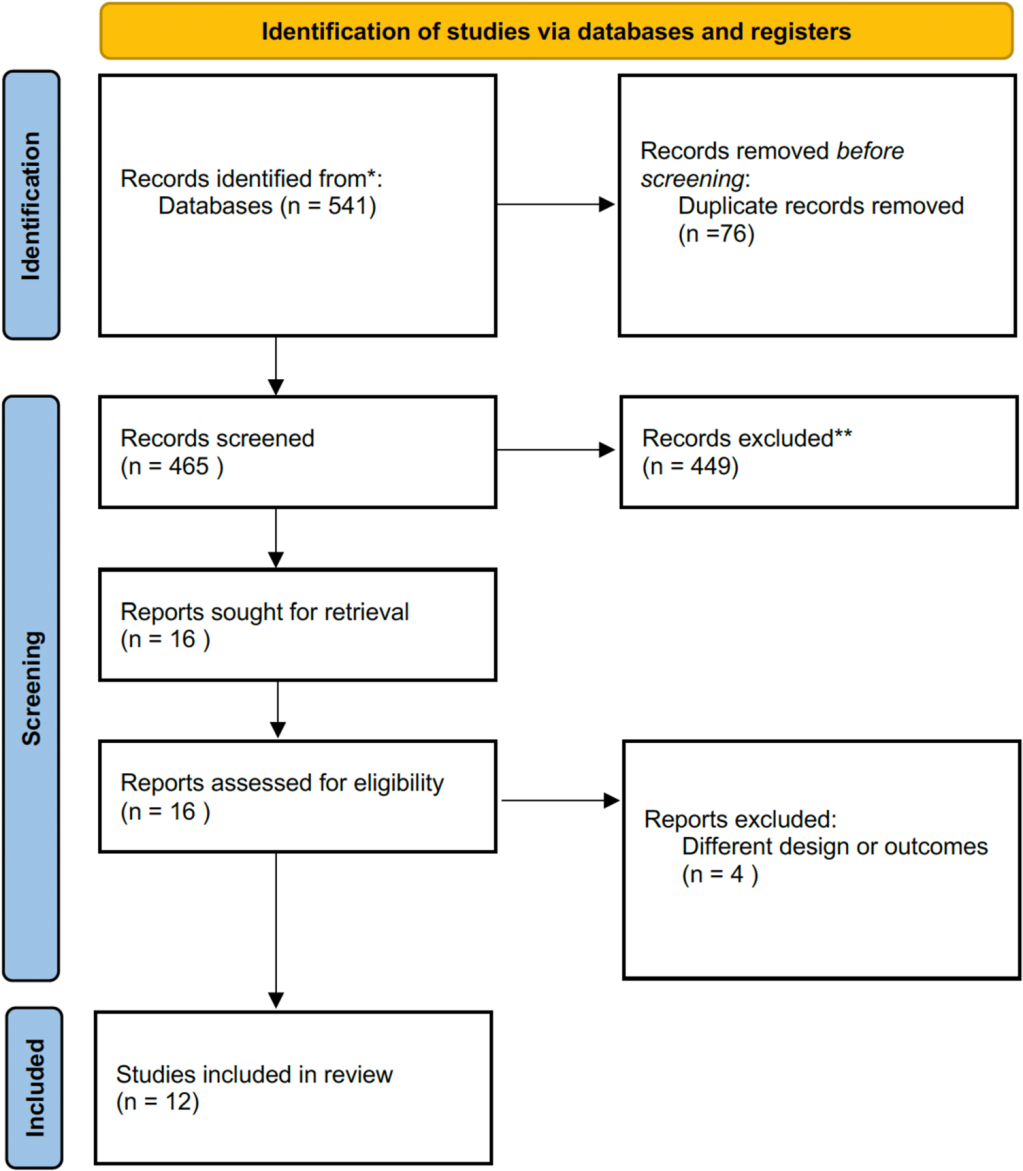
Flowchart of study screening according to PRISMA guidelines

The peer-reviewed literature published from January 1st, 2000 to August 1st, 2024 was searched using Medline (PubMed), Embase, Scopus, and Cochrane Library databases. The following key terms were used to identify relevant studies: “onlay”, “subcutaneous”, “endoscopic”, “onlay repair”, “subcutaneous onlay endoscopic approach”, “endoscopic onlay repair”, “laparoscopic”, “minimally invasive”, “robotic”, “hybrid”, “umbilical hernia”, “ventral hernia”, “epigastric hernia”, “incisional hernia”, “preaponeurotic”, “diastasis recti” and “rectus abdominis diastasis”. The detailed search strategies have been provided in the Supplementary Materials (Fig. 1S). This meta-analysis was reported in accordance with the Preferred Reporting Items for a Systematic Review and Meta-analysis (PRISMA) Statement [19]. The planned protocol of this meta-analysis was registered in PROSPERO (PROSPERO 2024: CRD42024573235). In addition, the reference lists of retrieved articles were screened to identify further studies.

### Study selection

Two investigators (FB, LB) independently performed the literature search and data extraction using Rayyan systematic review software [20]. They independently assessed the eligibility of all preliminary identified records based first on the title and then on the abstract. After preliminary selection, full-text manuscript of relevant studies was carefully read to confirm the eligibility and to extract useful information. Any disagreement regarding articles’ eligibility was solved by a third reviewer (LB). Studies were included according to the following criteria: (1) randomized and observational studies written in English, (2) adult population diagnosed with diastasis recti associated or not with primary or incisional ventral hernia, (3) repair performed using minimally invasive endoscopic onlay repair, (4) detailed description of the surgical technique and report of short-term outcomes. No geographic restrictions were applied. Reviews, editorials, case reports of < 5 patients and manuscripts regarding other minimally invasive diastasis recti and/or hernia repair techniques were excluded. Patients undergoing concomitant procedures were excluded. Papers were excluded if they reported duplicative results from the same authors’ group.

### Data extraction and quality assessment

Two authors examined the main features of each retrieved article, reporting the following data: year of publication, country where the study was performed and population source, total number of individuals, gender, age, outcomes, length of primary hospital stay (LOS), recurrence, length of follow-up, efficacy of the treatment performed, statistical analysis. The methodological quality of the selected studies was assessed using the Methodological Index for Non-randomized Studies (MINORS) criteria. The assessment of bias of included studies is reported into the Supplementary Materials (Fig. 1S). The overall quality of evidence was judged by means of the GRADE (Grading of Recommendations, Assessment, Development and Evaluations) approach [21]. Based on the overall assessment the quality was divided into four grades (high, moderate, low or very low). The studies were either downgraded or upgraded in quality depending on whether the criteria of risk of bias, inconsistency, indirectness, imprecision, publication bias, large magnitude, dose response or effect of all plausible confounding factors were met. Authors FB and LB performed the GRADE assessment.

### Outcome measures

Primary outcomes were evaluated based on safety and short-term measures, including:

- Intraoperative characteristics:
  Intraoperative complications, conversion rate and operative time
- Postoperative characteristics:
  Wound complications (surgical site infection, skin necrosis, hematoma, seroma), length of stay, recurrence rate, re-operation, readmission.

### Data synthesis and analysis

Meta-analysis was conducted using JAMOVI software [22]. Continuous data were expressed as means with standard deviations or medians with ranges, as appropriate. Also, categorical variables were shown as numbers and percentages for descriptive purposes. Statistical analysis was performed using both fixed- and random-effects models, depending on the level of heterogeneity across studies. For outcomes with high heterogeneity, a random-effects model was applied to account for variability between studies. The MAJOR package was used to pool means and proportions with 95% confidence intervals, respectively. A meta-analysis of binomial data using the score statistic and the exact binomial method, including the Freeman–Tukey double arcsine transformation of proportions, and stabilizing between-study variance was performed. Q and I2 statistics were used to test the heterogeneity among results. The low, moderate, and high degrees of heterogeneity correspond to I2 values of 25%, 50%, and 75% respectively. When high heterogeneity was detected, a sensitivity analysis was performed to assess the robustness of the overall findings by systematically excluding individual studies or subgroups to determine their impact on the pooled effect estimates.

## Results

### Search results

Search strategy retrieved 541 potentially eligible papers, restricted to 465 after removing duplicate records. After evaluation of titles and abstracts, further 449 records were excluded, resulting in 16 original articles preliminarily considered eligible for full-text examination. Among them 4 papers were excluded because of study design and outcomes. In the end, a total of 12 papers, including 480 patients, met all the inclusion criteria and were entered into the meta-analysis (publication dates 2015–2024). Figure 1 displays the 2020 PRISMA flowchart. General characteristics of the studies and the investigated groups are shown in Table 1. Results of the quality assessment of all included studies based on GRADE approach are shown in Supplementary Materials (Table 3S).

**Table 1:** Selected studies reporting the minimally invasive ENDOR approach

Author and year	Country	Enrollment	Type of publication	Total number of patients	MINORS score
Arias‑Espinosa et al., 2024 [25]	USA	2018–2023	Retrospective	15	14/16
Signorini et al., 2023 [23]	Argentina	2017–2019	Retrospective	54	14/16
Cuccomarino et al., 2022 [27]	Italy	2017–2019	Retrospective	124	9/16
Shinde et al., 2022 [29]	India	2020–2021	Prospective	30	14/16
Kler et al., 2020 [30]	UK	2018–2019	Retrospective	21	8/16
Gandhi et al., 2020 [26]	India	2016–2018	Retrospective	38	9/16
Dong et al., 2020 [28]	US	2018–2019	Prospective	16	15/16
Medina et al., 2019 [24]	Argentina	2014–2017	Prospective	42	11/16
Muas et al., 2019 [3]	Argentina	2014–2017	Prospective	48	15/16
Claus et al., 2018 [15]	Brasil	2015–2017	Retrospective	50	15/16
Barchi et al., 2018 [14]	Brazil	2016–2018	Prospective	21	15/16
Bellido Luque et al., 2015 [17]	Spain	2011–2012	Prospective	21	15/16

*MINORS* Methodological index for non-randomized studies

### Features of the retrieved studies

Among the twelve studies [3, 14, 15, 17, 23–30] included into the review, six of them were prospective [3, 14, 17, 24, 28, 29] and six retrospective [15, 23, 25–27, 30]. Preoperative and intraoperative patients’ characteristics are reported by Supplementary Table 1S. Among the 480 patients 133 (27.7%) were men and 347 (72.3%) women. The average age was 43.3 year-old (± 5.75) and the mean BMI 27.3 kg/m^2^ (± 0.7 kg/m^2^). Seven studies [15, 24–26, 28–30] reported hernia etiology, with 157 (80.92%) primary ventral hernias, 22 (11.34%) incisional hernias and 15 (7.73%) recurrent hernias. Hernia width was provided by seven studies [14, 15, 17, 25, 26, 28, 29], averaging 2.3 cm [± 1.29 cm] in size, while diastasis recti width by six of them [14, 15, 23–25, 27], averaging 3.33 cm [± 0.72] in size. Additional information on mesh placement is shown in Supplementary Table 1S.

### Meta-analysis

Nine studies [3, 14, 17, 23, 24, 26, 27, 29, 30] reported results of laparoscopic ENDOR, one of them [25] reported data of a totally robotic approach and two of them [15, 28] provided combined results of both robotic and laparoscopic approaches.

#### Intraoperative characteristics

**Fig. 2:**
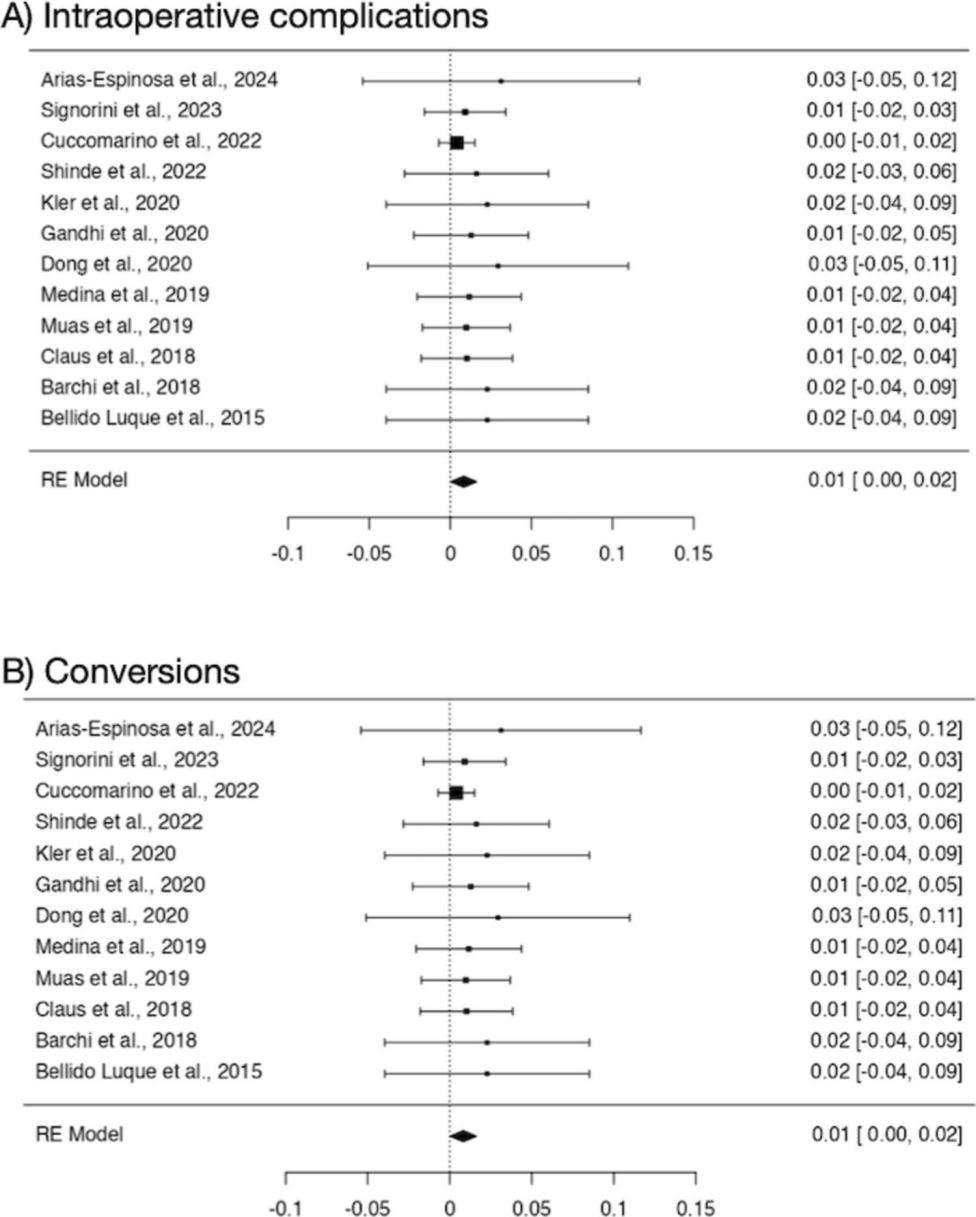
Forest plots of intraoperative characteristics

All studies analyzed intraoperative complications, with heterogeneity *p*-value of 0.998 and I2 value of 0%. The incidence of complications was 1%, with 95% confidence interval ranging from 0 to 2%. Similarly, conversion rate was assessed by all studies, with heterogeneity *p*-value of 0.991 and I2 value of 0%. The conversion rate was 1%, with a 95% confidence interval from 0 to 2%. Figure 2 depicts forest plots illustrating the intraoperative complication rate and conversion rate. The mean operative time was 103.14 min [95% CI 88.29–125.45].

#### Postoperative characteristics

**Fig. 3:**
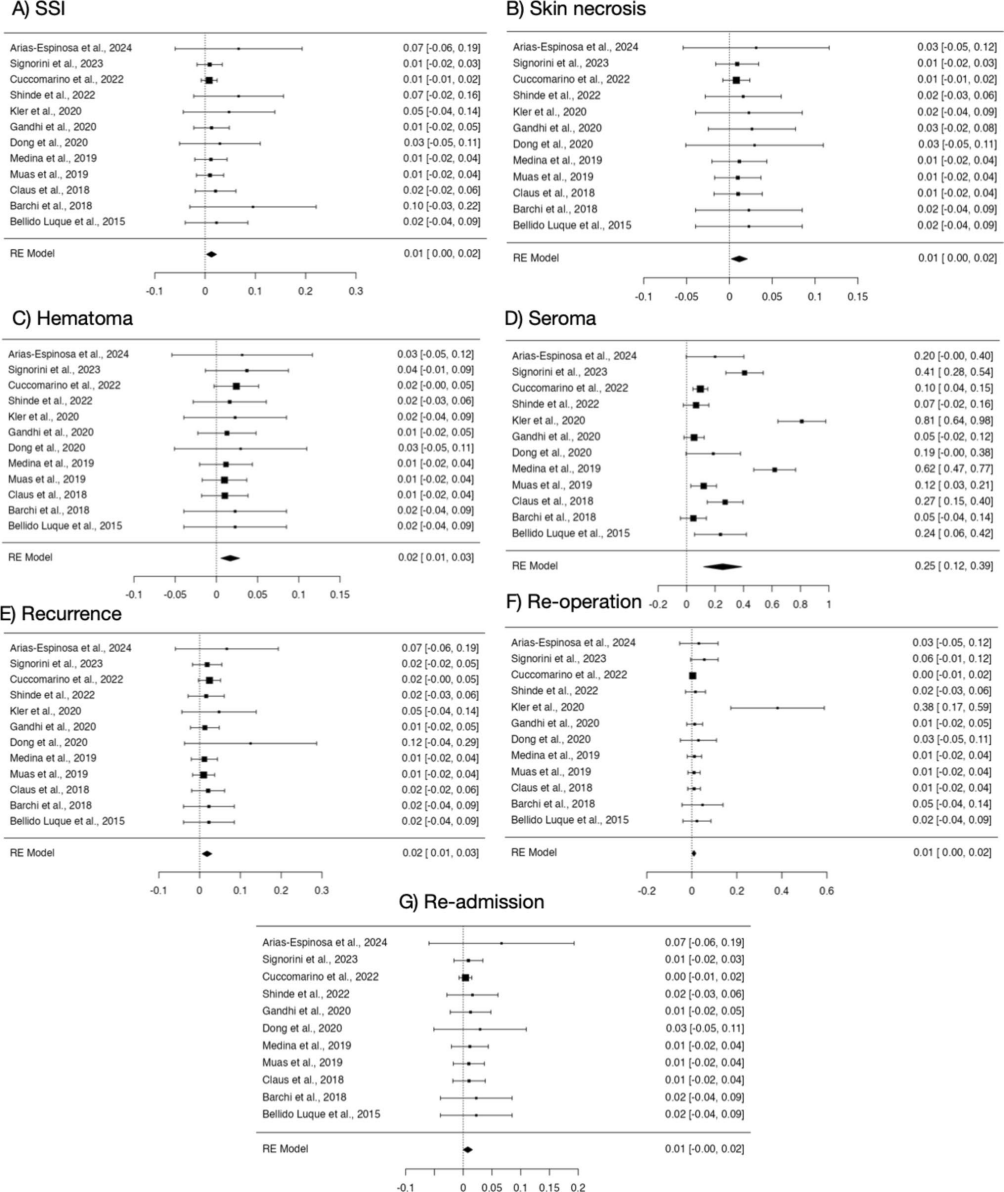
Forest plots of postoperative characteristics

All studies [3, 14, 15, 17, 23–30] reported surgical site infections (SSI) incidence (Fig. 3A), revealing low heterogeneity (*p*-value = 0.920; I2 = 0%). The pooled rate of SSI was 1% (95% CI 0–2%) as illustrated by Fig. 3A. Skin necrosis rate was 1% (95% CI 0–2%) (Fig. 3B). Additionally, hematoma rates were investigated by all studies [3, 14, 15, 17, 23–30], demonstrating low heterogeneity (*p*-value = 0.999; I2 = 0%) with pooled rate of 2% (95% CI 0.0–3%) (Fig. 3C). The incidence of seroma was examined by all studies [3, 14, 15, 17, 23–30], indicating high heterogeneity (*p*-value < 0.001; I2 = 94.9%) with pooled rate of 25% (95% CI 12–39%) (Fig. 3D). Given the high heterogeneity, a random-effects model was applied to this outcome as well and heterogeneity still resulted considerably high (I2 = 91% (Supplementary Materials, Fig. 2S). Through sensitivity analysis, it was observed that, excluding three studies [15, 23, 24] from the analysis, the I^2^ index decreased to 48.14%. This result suggests that these studies were major contributors to the overall variability. Indeed, after their removal, the overall seroma rate was 16% (95% CI 8–23%). Five studies [3, 14, 17, 24, 27] provided the median LOS, that was 1.3 days, ranging from 0.5 to 3 days. The median follow-up period was 16 months, ranging from 1.8 to 39 months. Recurrence rate was examined by all studies [3, 14, 15, 17, 23–30], revealing low heterogeneity (*p*-value = 0.982; I2 = 0%) with pooled rate of 2% (95% CI 1–3%) (Fig. 3E). Re-operation rates were also documented by all studies [3, 14, 15, 17, 23–30], showing low heterogeneity (*p*-value = 0.111; I2 = 0.03%) with pooled rate of 1% (95% CI 0–2%) (Fig. 3F). Moreover, readmission rates were provided by all studies [3, 14, 15, 17, 23–30], reflecting low heterogeneity (*p*-value = 0.993; I2 = 0%) with pooled rate of 1% (95% CI 0.0–3%) (Fig. 3G).

#### Methodological quality of studies

Studies [3, 14, 15, 17, 23–30] achieved a median MINORS score of 14/16 (range 10–15). This result suggests a moderate risk of bias across the included studies. The overall MINORS score evaluation for each study is shown in Table 1.

## Discussion

To our knowledge, this study is the first comprehensive systematic review and meta-analysis to rigorously evaluate the safety and short-term outcomes of the minimally invasive ENDOR procedure for diastasis recti and concurrent ventral hernia repair. According to our findings, the ENDOR technique is associated with low rates of intraoperative complications, re-operations, and conversions, each of them reaching 1% of incidence approximately. As regards short-term postoperative outcomes, the procedure demonstrates 1% incidence of SSI, 2% incidence of hematoma, 1% rate of hospital readmission and 25% incidence of seroma. The recurrence rate stands at 2%, with median follow-up duration of 16 months.

In this meta-analysis, the intraoperative complication and conversion rates associated with the ENDOR procedure were found to be negligible, underscoring the safety of this technique. These findings are consistent with outcomes reported for other surgical approaches, such as eTEP [31], LIRA [32], and TARUP [33]. Notably, ENDOR is associated with clear advantages regarding intraoperative safety, especially when compared to intraperitoneal repairs like IPOM [34]. Indeed, by avoiding direct contact with the viscera, the ENDOR approach significantly reduces the risk of iatrogenic injury [35, 36]. The average operative time for the ENDOR procedure is 103.14 min. Other minimally invasive approaches for diastasis recti and ventral hernia repair have a variable mean operative time, according to the technique. Aliseda et al. [31] report mean operative time of 148.89 min for eTEP repair, while shorter operative times have been observed with the LIRA and IPOM plus techniques, reported by Gómez-Menchero as 55.2 and 48.3 min, respectively [32]. Even if the time needed to complete the ENDOR procedure could seem long, this result reflects the variability of the surgeon’s experience and the learning curve associated with mastering this technique. Moreover, the longer operative times associated with the robotic approach contribute to the overall duration.

Postoperative complications such as surgical site infections, skin necrosis, and hematoma were infrequently reported by the analyzed studies. The ENDOR procedure has equivalent outcomes to other minimally invasive approaches. Laparoscopic and robotic techniques are preferred over the open ones due to their lower rates of surgical site infections and overall postoperative complications [34]. As a matter of fact, open procedures are associated with higher incidence of infections and complications, that significantly impacts LOS with consequent higher costs for the healthcare system [34]. While cutaneous necrosis remains a concern, it is rarely documented in the context of ENDOR, likely due to the preservation of lateral vascular structures. In our pooled analysis, the incidence of skin necrosis was negligible. However, two studies did report cases of cutaneous necrosis, which were successfully managed using negative pressure wound therapy (NPWT) [26, 27]. Hematoma formation was reported by two studies only, totalling five cases [23, 27]. The incidence of hematoma after the ENDOR procedure is comparable to the ones observed after the LIRA and IPOM plus techniques [32]. Although the eTEP technique might be associated with higher risk of hematoma [9], especially in the retro-muscular space, Aliseda et al. report a negligible hematoma rate for this approach in their meta-analysis [31].

The major concern of this procedure is the rate of seroma formation, that varies widely in literature, ranging from 12 to 47% [23]. Our pooled analysis reveals a postoperative seroma rate of 25% with high heterogeneity (I2 = 94.9%). Sensitivity analysis indicates that early removal of subcutaneous drainage (postoperative days 5–7) is a potential contributor to this variability, alongside differences in study design, population characteristics, and reporting practices. Excluding studies with early drain removal reduced heterogeneity and stabilized the pooled estimate, highlighting the robustness of our findings. Kler et al. [30] reported a high seroma rate of 81%, a finding that stands out when compared to other studies [3, 14, 26, 29]. This discrepancy may depend on some specific factors of their case series. Indeed, unlike most studies, in 57% of cases Kler et al. used biologic meshes, that may have influenced the high seroma rate observed. However, most seromas were managed conservatively. High BMI is associated with higher risk of seroma formation, likely due to the extensive subcutaneous dissection involved in the procedure, that creates a potential space for fluid collection [28]. This finding suggests that future research should analyze if the BMI correlates with the incidence of seroma formation and how. However, the variability of seroma incidence across studies may be influenced by differences in drainage use and time length it is left in place. Barchi et al. reported the lowest seroma rate, explaining these results by removing the drainage a median of 15 days after surgery [14]. Additionally, inconsistent use of postoperative compressive abdominal bandages across studies worsens data heterogeneity. Bellido Luque et al. highlighted that the use of abdominal bandages significantly reduced the seroma rate in the last cases of their series [17]. Comparatively, seroma rates among the other minimally invasive repair techniques are the following: 5% for eTEP [31], 6.9% for IPOM plus and 12% for IPOM [37]. The LIRA procedure had initially 40% incidence of radiological seromas, decreasing to 4.08% after one year of surgical practice [32].

Our systematic review identified eight cases of recurrence, even though data heterogeneity is high and it is influenced by the variety of prostheses, fixation methods and heterogeneous follow-up periods across the various studies. Indeed, Dong et al. [28] reported two recurrences in their series, both of them in patients who underwent surgery with the use of self-gripping mesh without any additional fixation, raising the ongoing debate over the need of supplementary fixation methods to prevent migration of this kind of mesh. Similarly, Signorini et al. noted that the first patient of their series suffered from recurrence, suggesting that this complication may result from the surgeon's early stage in the procedure's learning curve [23]. Recurrence incidence of diastasis recti and ventral hernia after other minimally invasive repair techniques is variable. The IPOM procedure has approximately 11.4% recurrence rate, while the IPOM plus technique around 6.6% [37]. The LIRA repair seems to be associated with favorable outcomes, as the recurrence rate is 0% at 15 months of follow-up [38]. However, this recurrence rate may be influenced by the limited sample sizes (68 patients from 2015 to 2022) and short follow-up periods [32]. The eTEP technique currently has around 1% recurrence rate, based on studies with 1–24 months follow-up periods [31].

The average length of hospital stay was 1.3 days, consistent with the ones reported for other minimally invasive diastasis recti and ventral hernia repair methods, both extra- and intraperitoneal [39, 40]. This length of stay is also significantly shorter if compared to the open repair techniques’ ones [34]. Additionally, Arias-Espinosa et al. reports that approximately 80% of the ENDOR surgeries were performed as outpatient procedures, highlighting the potential of reduced hospitalization associated with this approach [25].

The re-operation rate in this systematic review was 1%, that is consistent with the ones reported for other minimally invasive approaches of ventral hernia repair. For instance, Aliseda et al. also observed 1% re-operation rate in their study on the eTEP technique [31]. Nine out of the twelve re-operations recorded were due to chronic seroma, one of them was for recurrence, and two of them were for abdominal wall hematoma. Moreover, Signorini et al. reported statistically significant differences in the re-operation risk for chronic seroma between men and women [23]. These findings suggest that the technique may be safer for women, although further research is needed to fully understand the implications for men. Sex-related differences in peripheral adipose tissue may explain this trend. Women generally have more active abdominal fat lipolysis, that may lead to better subcutaneous fat preparation during surgery and, consequently, faster and more efficient recovery. This physiological advantage could reduce the likelihood of seroma formation in women, highlighting the need for sex-specific considerations in the management and prevention of postoperative complications.

Cosmetic outcomes are a key consideration in diastasis recti repair. While most studies reported favorable results, only Signorini et al. [23] objectively measured this using the EuraHS-QoL score, showing low aesthetic discomfort (mean 1.2, range 0–5) with no significant difference between sexes. Similarly, Medina et al. [24] reported a high average satisfaction score of 9.5 (range 8–10), with patients expressing that the procedure met their preoperative expectations. However, Medina et al. [24] did not use a standardized questionnaire, limiting comparability. These findings suggest high satisfaction with cosmetic outcomes, but the lack of consistent, validated tools across studies highlights the need for future research to systematically assess aesthetic and functional results using standardized measures like the EuraHS-QoL score.

As already highlighted by Muas et al. in their prospective study, this meta-analysis supports the use of endoscopic preaponeurotic surgery in patients affected by diastasis recti exceeding 3 cm, with or without concurrent midline hernia. Even though the width limit contraindicating this technique has not been defined yet, defects larger than 5 cm should be approached with caution. Indeed, these cases may require extensive lateral dissection, leading to potential skin bulging with consequent concern of patients seeking both functional and cosmetic outcomes [23]. The median hernia width reported in this meta-analysis was 2.3 cm (IQR 1.29 cm), indicating that the ENDOR technique has predominantly been used for relatively small ventral hernias. While this suggests its suitability for defects smaller than 5 cm, this threshold does not directly derive from the results but rather reflects the size range reported in the included studies. This work suggests that the ENDOR technique may be specifically indicated for symptomatic patients with diastasis recti associated with ventral hernias smaller than 5 cm in diameter and without excess skin and adipose tissue. These patients often suffer from low back pain, stress urinary incontinence, or dissatisfaction with the appearance of the abdominal wall. The technique appears to offer a balanced approach for addressing both functional and aesthetic concerns of these patients. In patients with excess skin and adipose tissue open abdominoplasty remains an excellent option, offering the dual benefits of soft tissue excision and rectus muscle plication, it is not suitable for all patients. Minimally invasive techniques, such as the ENDOR approach, provide an alternative for individuals without significant skin excess or those seeking to avoid the large incisions associated with open procedures.

This study has several limitations, primarily due to the low level of evidence available for the ENDOR technique. Most included studies were observational, making them prone to selection bias and potentially overestimating benefits. The GRADE assessment rated the quality of evidence for most outcomes as low, with the seroma rate rated as very low due to high heterogeneity and potential reporting bias. Publication bias analysis was limited by the small number of studies per outcome and the rarity of certain events, restricting the reliability of funnel plots and statistical tests. Although sensitivity analyses and qualitative assessments were conducted, the findings must be interpreted cautiously. Additional limitations include the lack of randomized controlled trials, heterogeneity in follow-up durations, and inconsistent definitions and reporting of key outcomes such as SSI, conversions, and re-operations. Another important limitation is the influence of surgical expertise. The included procedures were performed by highly experienced surgeons, many of them pioneers of the ENDOR approach, raising concerns about the generalizability of these results to less experienced surgeons or centers. While the findings are promising, more robust and standardized prospective studies are needed to confirm the safety and efficacy of ENDOR.

This systematic review and meta-analysis offers a comprehensive summary of the intraoperative and short-term postoperative outcomes associated with minimally invasive ENDOR procedure. The findings suggest that this technique is a safe and feasible approach for diastasis recti and ventral hernia repair, when performed by expert abdominal wall surgeons, but the debate regarding pre-aponeurotic mesh placement and its impact on recurrence and seroma formation is still open. However, to fully evaluate its efficacy and safety, randomized clinical trials with longer follow-up periods are needed to assess both its short- and long-term outcomes including abdominal wall function, tissue cosmesis, and bulging.

## Supplementary Information

Below is the link to the electronic supplementary material.

Supplementary file1 (DOCX 326 KB)
