# Supplementary material for: Short‑term outcomes of minimally invasive endoscopic onlay repair for diastasis recti and ventral hernia repair: a systematic review and meta‑analysis
Source: Surg Endosc. 2025 Feb 7;39(3):1490–500. doi: 10.1007/s00464-025-11555-1 (PMC11870909; doi:10.1007/s00464-025-11555-1)
Supplement: Supplementary file 1 — Supplementary file1 (DOCX 326 KB) [file 464_2025_11555_MOESM1_ESM.docx]

**Figure 1s: detailed search strategy**

**Medline**

(("Subcutaneous OnLay" OR "Endoscopic OnLay" OR "robotic OnLay" OR "minimally invasive OnLay")

AND

("ventral hernia" OR "umbilical hernia" OR "diastasis recti"))

**Scopus**

TITLE-ABS(("Subcutaneous OnLay" OR "Endoscopic OnLay" OR "robotic OnLay" OR "minimally invasive OnLay")

AND

("ventral hernia" OR "umbilical hernia" OR "diastasis recti"))

**Embase**

('subcutaneous onlay':ab,ti OR 'endoscopic onlay':ab,ti OR 'robotic onlay':ab,ti OR 'minimally invasive onlay':ab,ti)

AND

('ventral hernia'/exp OR 'umbilical hernia'/exp OR 'diastasis recti'/exp)

**Cochrane Library**

("Subcutaneous OnLay" OR "Endoscopic OnLay" OR "robowtic OnLay" OR "minimally invasive OnLay")

AND

("ventral hernia" OR "umbilical hernia" OR "diastasis recti")

**Figure 2s: Seroma rate, Random-effect model**

**
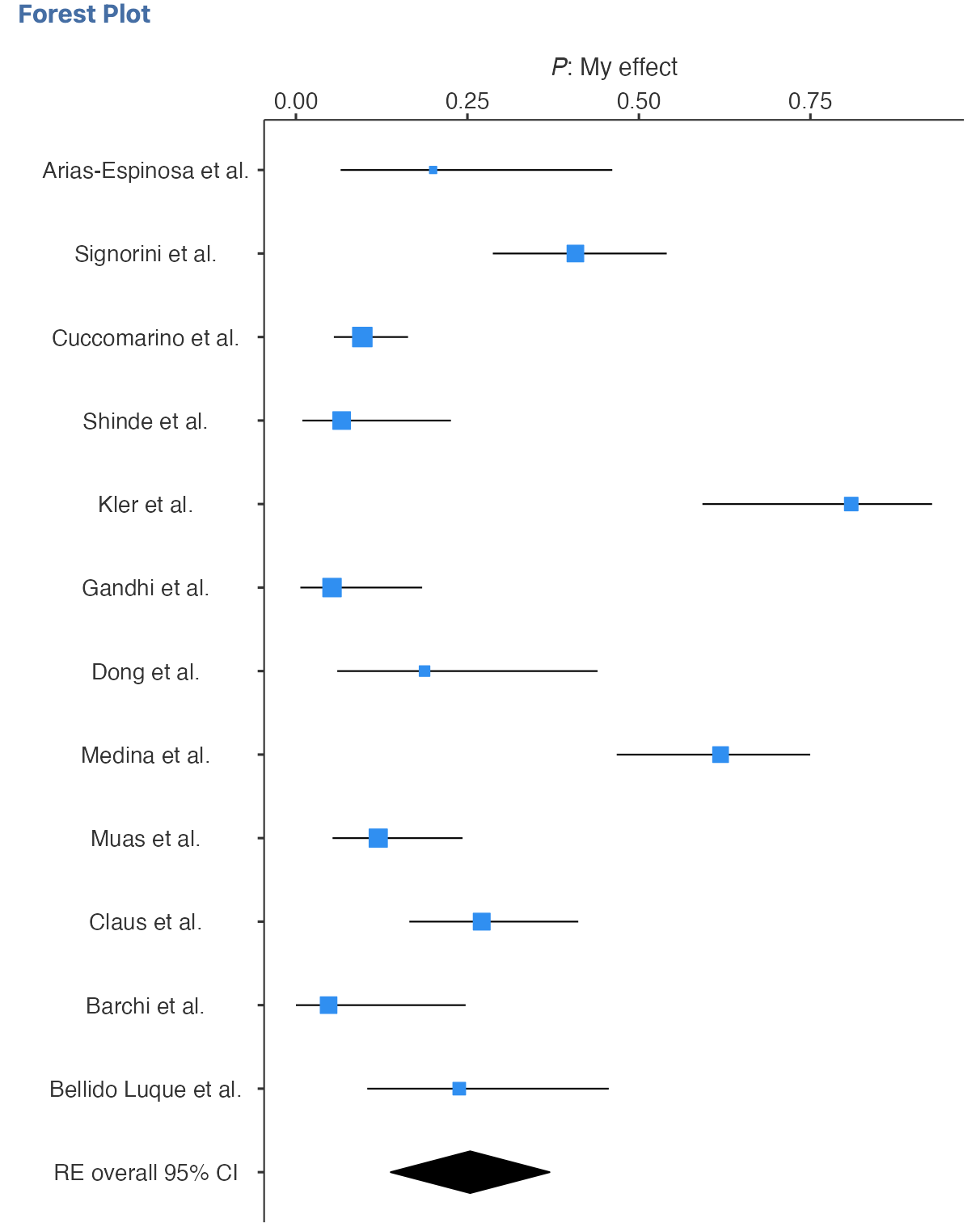
**

**
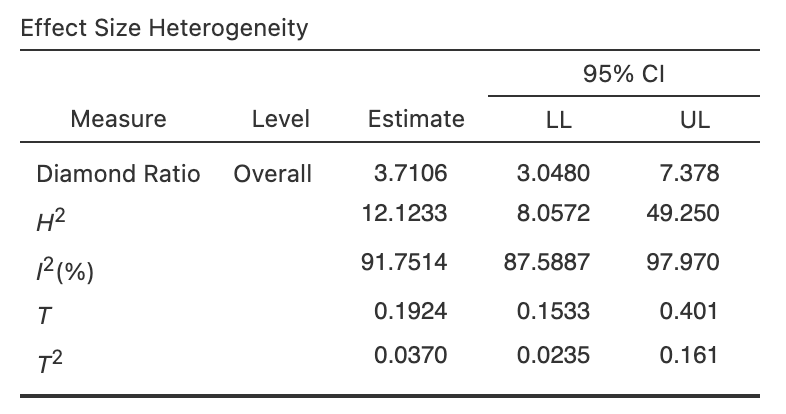
**

**Figure 3s: Funnel plot for SSI rates**
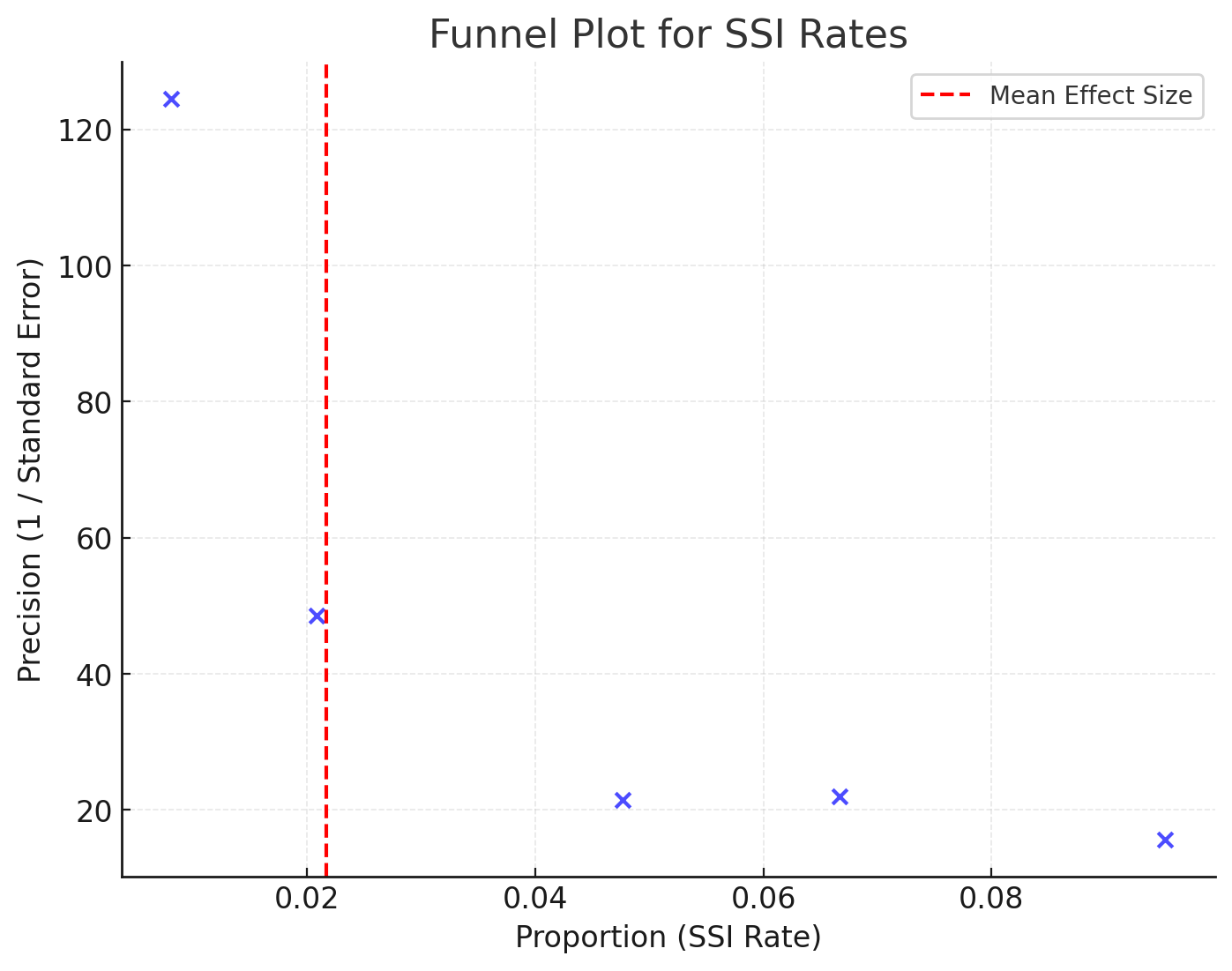


**Figure 4s: Funnel plot for Hematoma rates**

**
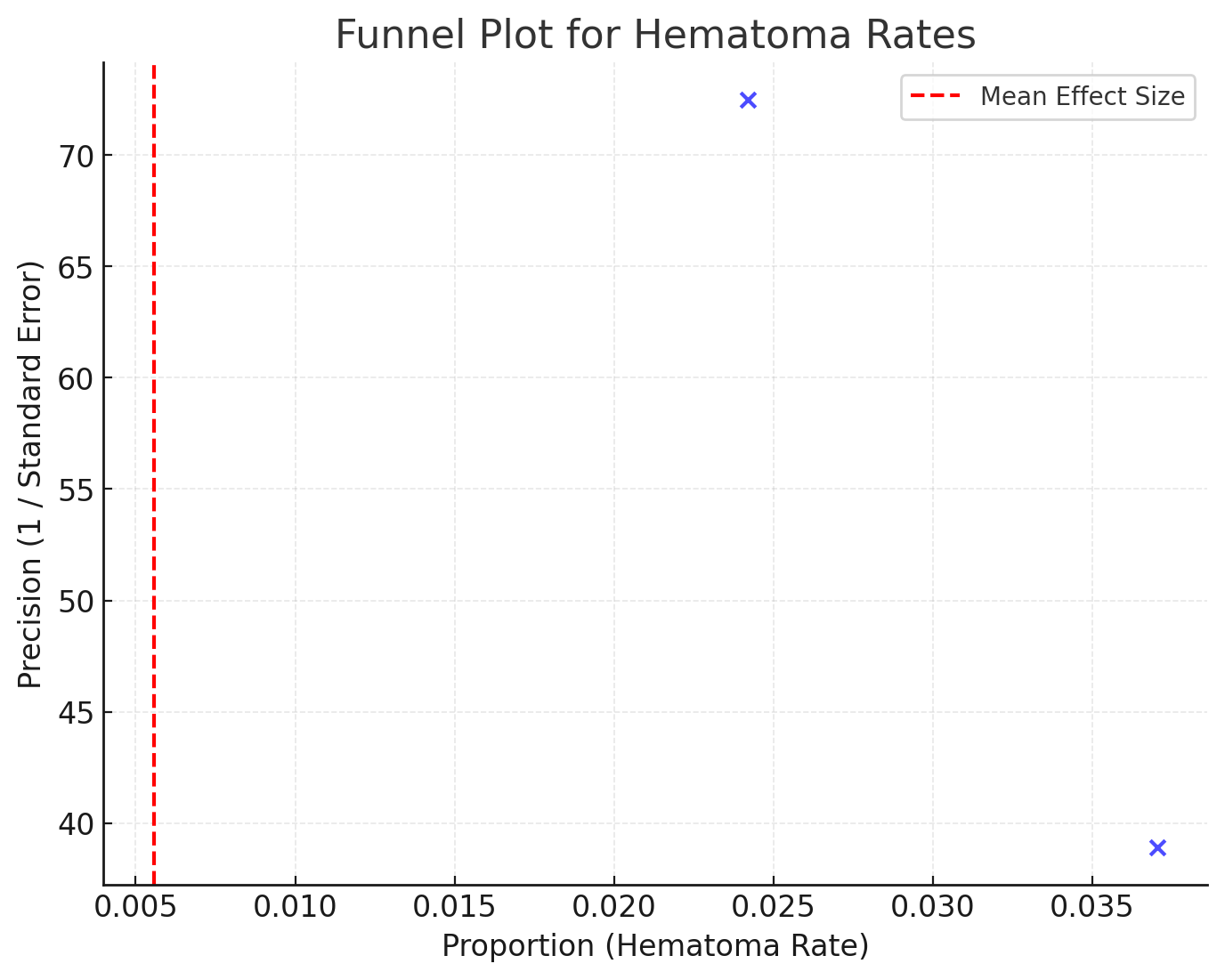
**

**Figure 5s: Funnel plot for Seroma rates**

**
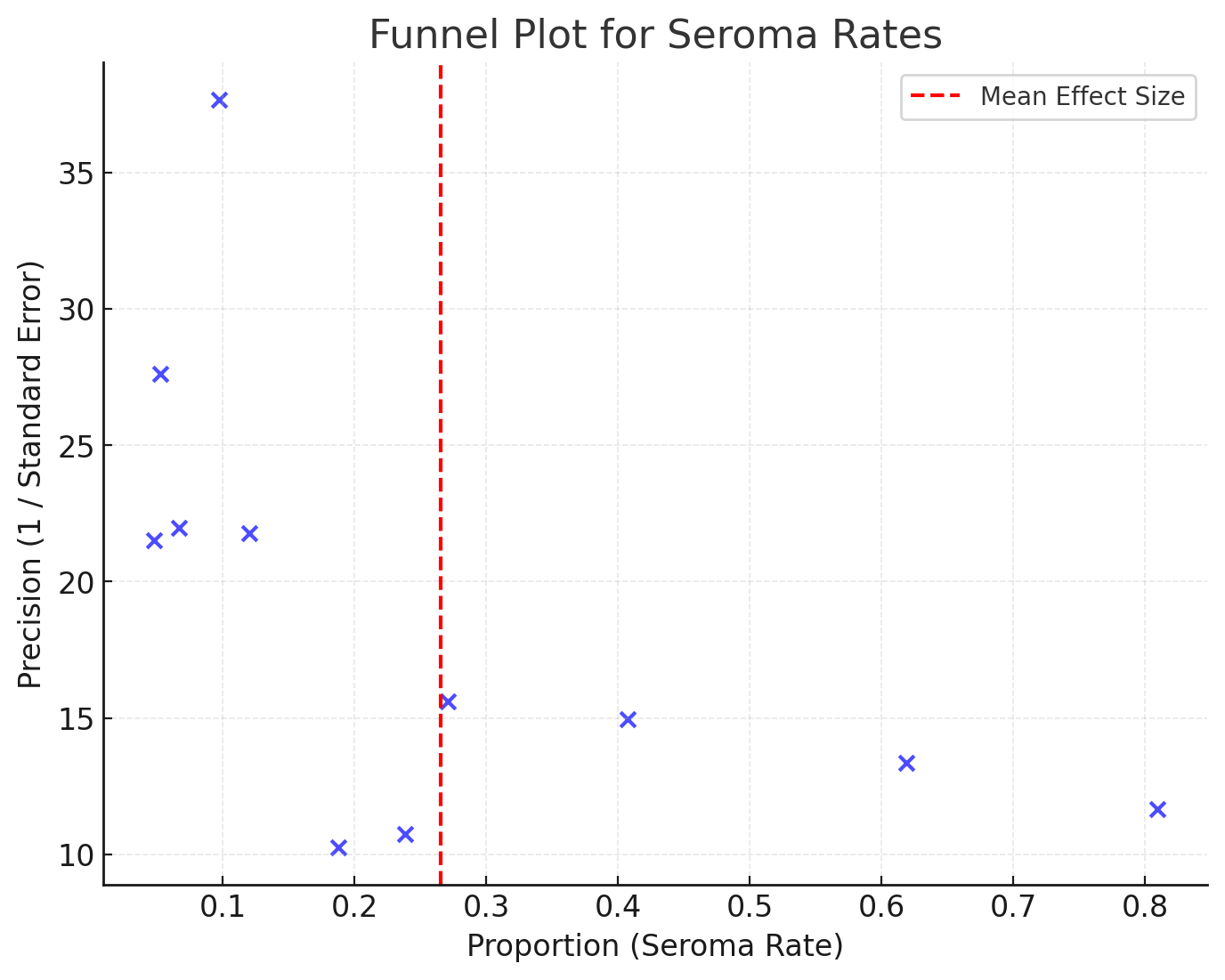
**

**Figure 6s: Funnel plot for Recurrence rates**


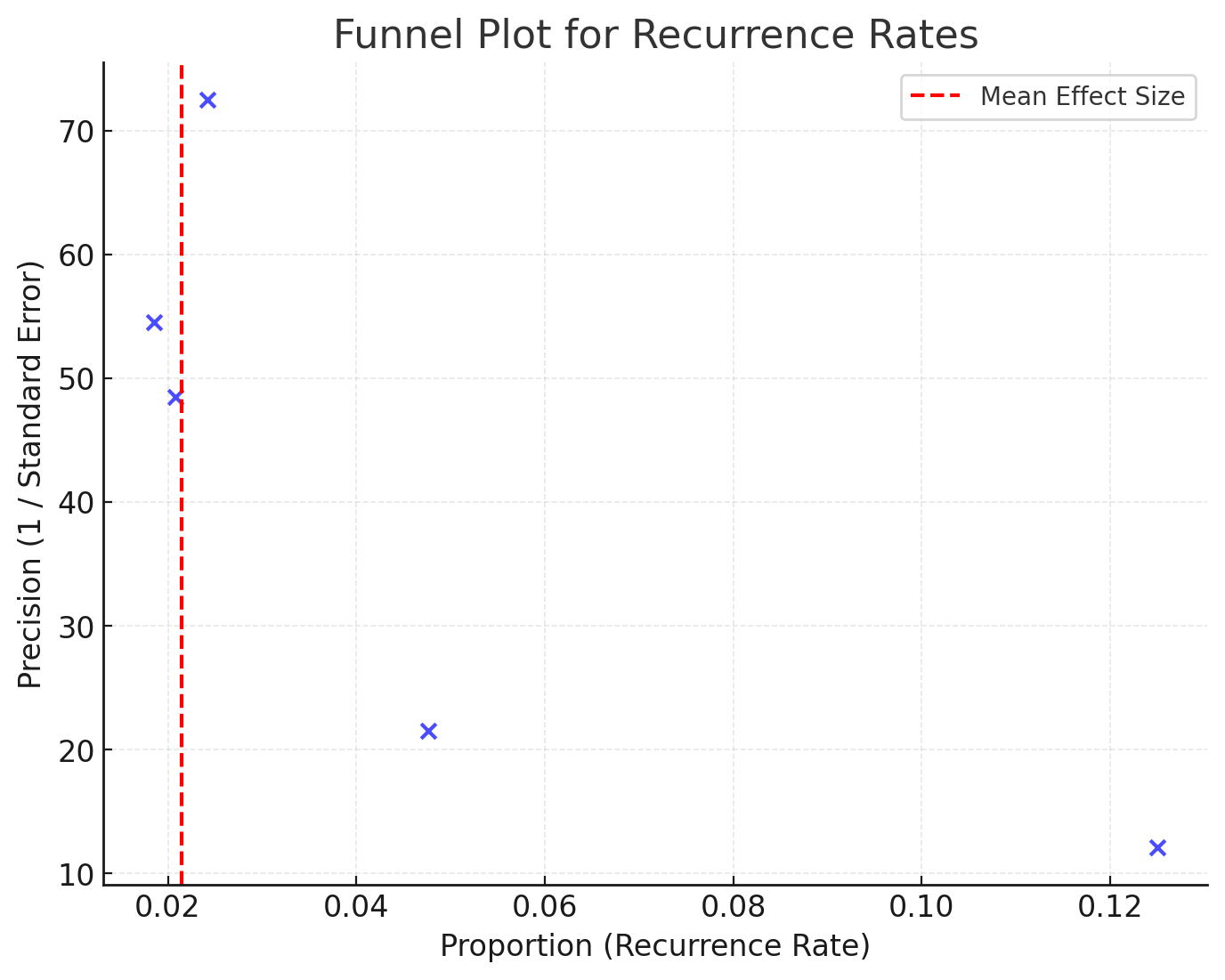


**Figure 7s: Funnel plot for Reoperation rates**


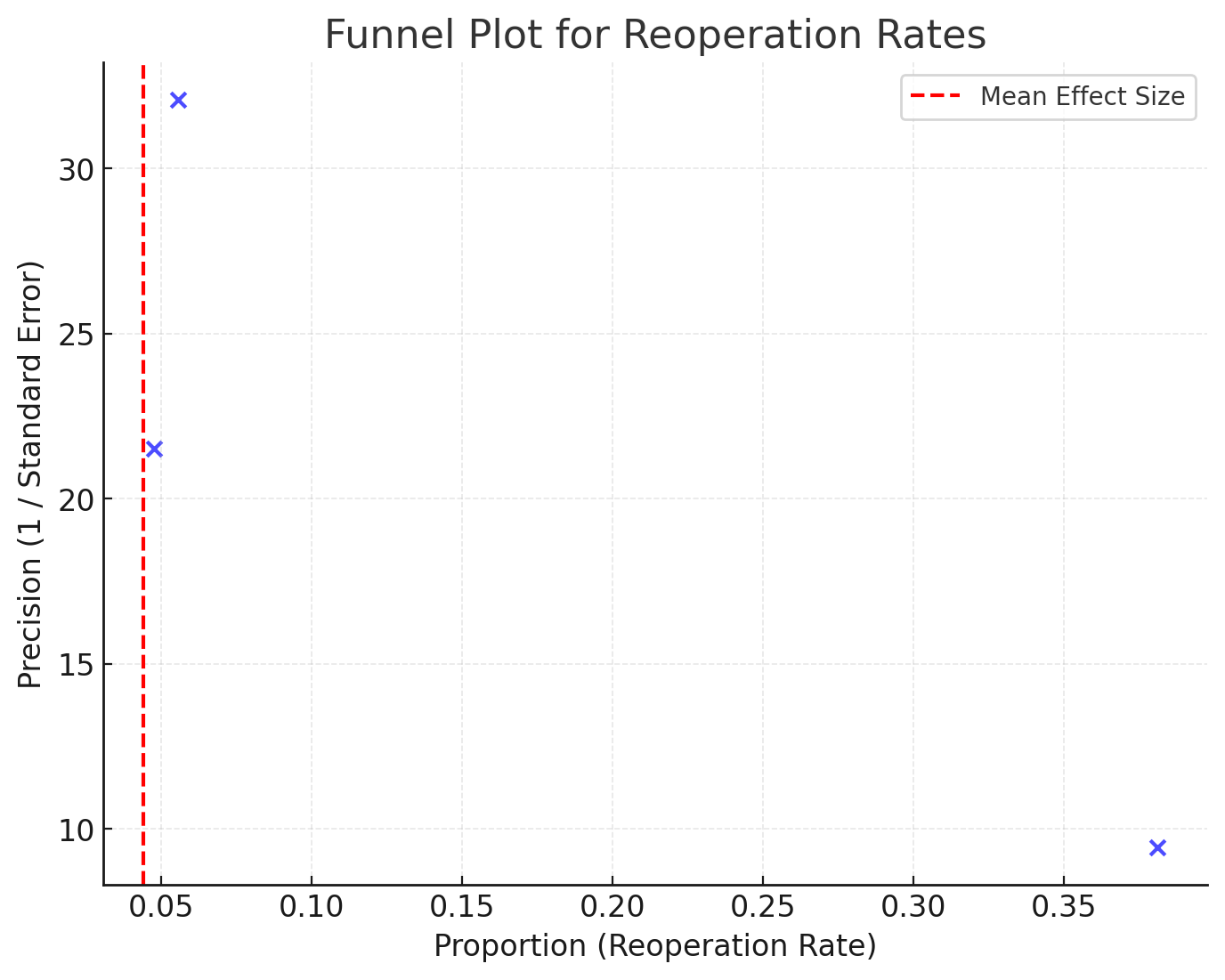


**Table 1s:** Preoperative and intraoperative patient characteristics

| **Author and year** | **Age** | **Sex ratio M/F (%)** | **BMI (kg/m2)** | **Primary/Incisional/Recurrent umbilical hernia (%)** | **Technique Laparo/Robot (%)** |
| --- | --- | --- | --- | --- | --- |
| Arias‑Espinosa et al., 2024 | 59 (42–63)* | 6 (40) /9 (60) | 25.4 (21.7–33.8)* | 7 (46.6)/ 2 (13.4)/0 | 0 (0)/ 15 (100) |
| Signorini et al., 2023 | 50.7 ± 54^☨^ | 29 (53.7)/25 (46.3) | 28.7 ± 20^☨^ | NR | 54 (100)/0 (0) |
| Cuccomarino et al., 2022 | 42 (29–65)* | 6 (4.8%)/118 (95.2%) | 22.5 (17.6–30.5)* | NR | 124 (100)/0 (0) |
| Shinde et al., 2022 | 42,3^☨^ | 20 (66.66)/10 (33.33) | 28.9^☨^ | 30 (100)/0/0 | 30 (100)/(0) |
| Kler et al., 2020 | 53 (33-77)* | 8 (38.1) / 13 (62.9) | 29.7* | NR/NR/3 | 21 (100)/0 (0) |
| Gandhi et al., 2020 | 42 (35 - 52)* | 14 (36.8) /24 (63.2) | 28.3 (25.1-33)* | 30 (78.9)/8 (21.1) | 38 (100)/ 0 (0) |
| Dong et al., 2020 | 45.7 ± 11.9^☨^ | 2 (12.5)/14 (87.5) | 29 ± 3.6^☨^ | 12 (68.7)/2 (12.5)/2 (18.8) | 14 (87.5)/2 (12.5) |
| Medina et al., 2019 | 39 (26-63)* | 10 (24) / 32 (76) | 26.8 (21.6-28.6)* | 41 (80.4)/10 (19.6)/0 | 42 (100)/0 (0) |
| Muas et al., 2019 | 44.25 ± 29^☨^ | 20 (41.7)/28 (58.3) | 27.7 ± 10^☨^ | 38 (79)/0/10 (21) | 44 (91.6)/4 (8.4) |
| Claus et al., 2018 | 38 ± 42^☨^ | 3 (6)/47 (94) | NR | NR | 50 (100)/0 (0) |
| Barchi et al., 2018 | 47.5 ± 33^☨^ | 12 (57.2)/9 (42.8) | 26.3 ± 14^☨^ | NR | 21 (100)/0 (0) |
| Bellido Luque et al., 2015 | 37.6 ± 26^☨^ | 3 (14.3)/18 (85.7) | 27.4 ± 13^☨^ | NR | 21 (100)/0 (0) |

(continues…)

| **Author and year** | **Diastasis width (cm)** | **Hernia defect (width cm)** | **Mesh type** | **Mesh area (cm2)** | **Mesh lenght (cm)** | **Mesh width (cm)** | **Drain** |
| --- | --- | --- | --- | --- | --- | --- | --- |
| Arias‑Espinosa et al., 2024 | 4 (3–6)* | 2 (2–2.25)* | Synthetic | NR | 23 (20–28)* | 11.5 (10–16)* | 19-French Blake® drain and removed in 10–14 days. |
| Signorini et al., 2023 | 2.6 ± 2.8^☨^ | NR | Synthetic | NR | 14.83 ± 2.47^☨^ | 12.59 ± 2.5^☨^ | silicone drain, removed in 5-7 days |
| Cuccomarino et al., 2022 | 5.22 (2.5–15)* | NR | Synthetic | 337.5 | 20* | 15 | NR |
| Shinde et al., 2022 | NR | 2,1^☨^ | Synthetic | NR | NR | NR | Two subcutaneous Jackson-Pratt drains. Kept till the output is below 30 ml |
| Kler et al., 2020 | NR | NR | Biologic or Synthetic | NR | NR | NR | 2 suction drains, removed in 3 days |
| Gandhi et al., 2020 | NR | 3.8 (1.5 - 3.9)* | Synthetic | NR | NR | NR | Flat drain removed after output reduced to 10 ml/day. |
| Dong et al., 2020 | NR | 1.9 ± 0.7^☨^ | Synthetic | NR | NR | NR | 19 French round drain removed after output reduced to 50 ml/day. |
| Medina et al., 2019 | 5.5 (4-7)* | NR | Synthetic | 130 | 13* | 10 | drain, removed in 5-7 days |
| Muas et al., 2019 | 4.05 ± 3^☨^ | 2.3 ± 2.5^☨^ | Synthetic | NR | NR | NR | Suction drain ,  with a 3.68 ± 1.8 days permanence  Removed when the remaining was  < 50 cc/day. |
| Claus et al., 2018 | NR | NR | Synthetic | NR | NR | NR | NR |
| Barchi et al., 2018 | 3.2 ± 1.5^☨^ | 3.27 ± 5.8^☨^ | Synthetic | 453.3 ± 271^☨^ | NR | 17.6 ± 7^☨^ | removing the drain with a median of 15 days |
| Bellido Luque et al., 2015 | NR | 3.4 ± 0.78^☨^ | Synthetic | NR | NR | NR | NR |

**M= male; F= female; * median (range); ☨ mean** ± standard deviation; NR = not reported; BMI = body mass index

**Table 2s:** MINORS score evaluation

| **Author and year** | **A clearly stated aim** | **Inclusion of consecutive patients** | **Prospective collection of data** | **Endpoints appropriate to the aim of the study** | **Unbiased assessment of the study endpoint** | **Follow-up period appropriate to the aim of the study** | **Loss to follow up less than 5%** | **Prospective calculation of the study size** | **An adequate control group** | **Contemporary groups** | **Baseline equivalence of groups** | **Adequate statistical analyses** | **Total score** |
| --- | --- | --- | --- | --- | --- | --- | --- | --- | --- | --- | --- | --- | --- |
| Arias‑Espinosa et al., 2024 | 2 | 1 | 1 | 2 | 1 | 2 | 2 | 1 | 0 | 0 | 0 | 2 | **14** |
| Signorini et al., 2023 | 2 | 1 | 1 | 2 | 1 | 2 | 2 | 1 | 0 | 0 | 0 | 2 | **14** |
| Cuccomarino et al., 2022 | 2 | 0 | 1 | 1 | 1 | 1 | 1 | 1 | 0 | 0 | 0 | 1 | **9** |
| Shinde et al., 2022 | 1 | 1 | 2 | 2 | 1 | 2 | 2 | 1 | 0 | 0 | 0 | 2 | **14** |
| Kler et al., 2020 | 1 | 0 | 1 | 1 | 1 | 1 | 1 | 1 | 0 | 0 | 0 | 1 | **8** |
| Gandhi et al., 2020 | 2 | 0 | 1 | 1 | 1 | 1 | 1 | 1 | 0 | 0 | 0 | 1 | **9** |
| Dong et al., 2020 | 2 | 1 | 2 | 2 | 1 | 2 | 2 | 1 | 0 | 0 | 0 | 2 | **15** |
| Medina et al., 2019 | 2 | 0 | 2 | 1 | 1 | 2 | 1 | 1 | 0 | 0 | 0 | 1 | **11** |
| Muas et al., 2019 | 2 | 1 | 2 | 2 | 1 | 2 | 2 | 1 | 0 | 0 | 0 | 2 | **15** |
| Claus et al., 2018 | 2 | 1 | 2 | 2 | 1 | 2 | 2 | 1 | 0 | 0 | 0 | 2 | **15** |
| Barchi et al., 2018 | 2 | 1 | 2 | 2 | 1 | 2 | 2 | 1 | 0 | 0 | 0 | 2 | **15** |
| Bellido Luque et al., 2015 | 2 | 1 | 2 | 2 | 1 | 2 | 2 | 1 | 0 | 0 | 0 | 2 | **15** |

Table 3s: GRADE approach for included studies

| Outcome | N. of studies | Study design | Risk of bias | Inconsistency | **Indirectness** | **Imprecision** | **Publication Bias** | GRADE quality  of evidence |
| --- | --- | --- | --- | --- | --- | --- | --- | --- |
| Intraoperative complications | 12 | Observational | Downgraded 1: Risk of bias due to non-standardized reporting | No downgrade: Low heterogeneity ( I2 = 0%). | No downgrade: Directly relevant to research question. | No downgrade: Pooled estimate has narrow CIs | Not Downgraded : No evidence of publication bias observed; all included studies reported a 0% readmission rate, limiting variability and precluding funnel plot analysis | Low |
| Conversions | 12 | Observational | Downgraded 1: Variability in reporting of conversion criteria across studies. | No downgrade: Low heterogeneity ( I2 = 0%). | No downgrade: Directly relevant to research question. | No downgrade: Pooled estimate has narrow CIs | Not Downgraded : No evidence of publication bias observed; all included studies reported a 0% readmission rate, limiting variability and precluding funnel plot analysis | Low |
| SSI | 12 | Observational | Downgraded 1: Variability in definition and reporting of SSI. | No downgrade: Low heterogeneity ( I2 = 0%). | No downgrade: Directly relevant to research question. | No downgrade: Pooled estimate has narrow CIs | Not downgraded: No strong evidence of publication bias observed. Funnel plot asymmetry is minimal and likely reflects the rarity of SSIs | Low |
| Skin necrosis | 12 | Observational | Downgraded 1: Risk of bias in outcome reporting across studies. | No downgrade: Low heterogeneity ( I2 = 0%). | No downgrade: Directly relevant to research question. | No downgrade: Pooled estimate has narrow CIs | Not Downgraded : No evidence of publication bias observed; all included studies reported a 0% readmission rate, limiting variability and precluding funnel plot analysis | Low |
| Hematoma | 12 | Observational | Downgraded 1: Risk of bias in outcome reporting across studies. | No downgrade: Low heterogeneity ( I2 = 0%). | No downgrade: Directly relevant to research question. | No downgrade: Pooled estimate has narrow CIs | Not downgraded: No strong evidence of publication bias observed. Funnel plot asymmetry is minimal and likely reflects the rarity of hematoma events | Low |
| Seroma | 12 | Observational | Downgraded 1: Some studies with moderate bias (e.g., early drain removal not standardized). | Downgraded 1: High heterogeneity ( I2 = 94.9%, sensitivity analysis reduced to 48.14%). | No downgrade: Directly relevant to research question. | Downgraded 1: Wide CIs around pooled estimate. | No downgrade: Funnel plot does not suggest publication bias | Very Low |
| Recurrence | 12 | Observational | Downgraded 1: Moderate methodological quality (e.g., short follow-up durations). | No downgrade: Low heterogeneity ( I2 = 0%). | No downgrade: Directly relevant to research question. | No downgrade: Pooled estimate has narrow CIs | Not downgraded No strong evidence of publication bias observed. Funnel plot asymmetry is minor and likely reflects the rarity of recurrence events. Given the limited number of studies, formal tests for publication bias were not feasible | Low |
| Re-operation | 11 | Observational | Downgraded 1: Risk of bias due to incomplete follow-up in some studies | No downgrade: Low heterogeneity ( I2 = 0%). | No downgrade: Directly relevant to research question. | No downgrade: Pooled estimate has narrow CIs | Not downgraded: No strong evidence of publication bias observed. Funnel plot asymmetry likely reflects the rarity of reoperations rather than selective reporting. Due to the low number of studies with non-zero events, formal tests for publication bias were not feasible.. | Low |
| Re-admission | 11 | Observational | Downgraded 1: Risk of bias due to incomplete reporting in some studies | No downgrade: Low heterogeneity ( I2 = 0%). | No downgrade: Directly relevant to research question | No downgrade: Pooled estimate has narrow CIs | Not Downgraded : No evidence of publication bias observed; all included studies reported a 0% readmission rate, limiting variability and precluding funnel plot analysis | Low |
